# Evaluation of the Direct Economic Cost per Eradication Treatment Regimen against *Helicobacter pylori* Infection in Greece: Do National Health Policy-Makers Need to Care?

**DOI:** 10.3390/medicina56030133

**Published:** 2020-03-18

**Authors:** Christos Liatsos, Apostolis Papaefthymiou, Nikolaos Kyriakos, Marios Giakoumis, Jannis Kountouras, Michail Galanopoulos, Periklis Apostolopoulos, Sotirios D. Georgopoulos, Christos Mavrogiannis, Aristomenis K. Exadaktylos, David Shiva Srivastava, Theodore Rokkas, Michael Doulberis

**Affiliations:** 1 Department of Gastroenterology, 401 Army General Hospital of Athens, 11525 Athens, Greece; appapaef@hotmail.com (A.P.); nikos_kiriakos@yahoo.gr (N.K.); giakoumismarios@hotmail.com (M.G.); galanopoulosdr@gmail.com (M.G.); 2 Department of Medicine, Second Medical Clinic, Aristotle University of Thessaloniki, Ippokration Hospital, 54642 Thessaloniki, Greece; jannis@auth.gr (J.K.); doulberis@gmail.com (M.D.); 3 Department of Gastroenterology, Army Share Fund Hospital (NIMTS), 11521 Athens, Greece; periklisapo@yahoo.com; 4 Department of Gastroenterology, Athens Medical, Paleo Faliron Hospital, 17562 Palaio Faliro, Greece; georgpap@ath.forthnet.gr; 5 Gastroenterology & Liver Unit, National and Kapodistrian University of Athens, General & Oncology Hospital of Kifissia “Agioi Anargyroi”, 14564 Kifissia, Greece; cmavrog@nurs.uoa.gr; 6 Department of Emergency Medicine, University Hospital Inselspital Bern, 3010 Bern, Switzerland; aristomenis.exadaktylos@insel.ch (A.K.E.); david.srivastava@insel.ch (D.S.S.); 7 Gastroenterological Clinic, Henry Dunant Hospital, 11526 Athens, Greece; sakkor@otenet.gr; 8 Department of Gastroenterology and Hepatology, University of Zurich, 8091 Zurich, Switzerland

**Keywords:** *Helicobacter pylori*, *Hp* infection, treatment cost, eradication regimens

## Abstract

*Helicobacter pylori* (*Hp*) management has undoubtedly resulted in a notable economic burden on healthcare systems globally, including Greece. Its cost has never been estimated so far, especially during the recent 10-year unprecedented financial crisis. Direct medical and procedural costs for one attempt “outpatient” *Hp* eradication treatment were estimated as the following: (I) first-line regimens: 10 and 14 days standard triple, 10 and 14 days sequential, 10 and 14 days concomitant non-bismuth quadruple, 14 days hybrid, (II) second-line salvage regimens: 10 and 14 days levofloxacin-containing triple regimens. Treatment costs using prototypes and/or generic drugs were calculated. Drug prices were collected and confirmed from two official online medical databases including all medicines approved by the Greek National Organization for Medicines. Regimens based on generics were more affordable than prototypes and those including pantoprazole yielded the lowest prices (mean: 27.84 €). Paradoxically, 10-day concomitant and 14-day hybrid regimens (currently providing good (90–94%) first-line eradication rates in Greece) cost the same (mean: 34.76 €). The expenditures for *Hp* eradication treatment regimens were estimated thoroughly for the first time in Greece. These data should be taken into account by Public Health policymakers both in Greece and the European Union, aiming for a better and less expensive therapeutic approach.

## 1. Introduction

Greece has been experiencing a financial decline during the last decade, which is a recognizable representative of the global crisis, and the resultant austerity policies have affected national healthcare expenditures [1,2]. A 35% reduction in the National Health System resources was noted between the onset of the crisis and 2016; concurrently, spending for health was among the lowest in Europe [3]. This reduction, at least at the beginning, did not affect medication costs, a fact that is quite obvious considering that in 2013, proton pump inhibitors (PPIs) in Greece were among the most expensive drugs compared to other European countries (similar to Switzerland) [4]. Additionally, the prescription of generics was sluggish in Greece until it became compulsory for both doctors and pharmacists to provide patients with the most affordable choices [4,5].

*Helicobacter pylori* infection (*Hp-*I) affects 40.2–64.0% of the Greek population, following the global prevalence and an increasing tendency of infectious diseases in our country attributed to economic hardships and migratory flows [6,7,8,9]. According to current guidelines [10], peptic ulcer disease, gastritis, gastric atrophy, gastric cancer, gastric mucosa-associated lymphoid tissue lymphoma, idiopathic thrombocytopenic purpura or iron-deficiency anemia necessitate a *Hp* test and treat strategy, whereas a plethora of further disorders have been connected with *Hp*-I, such as colorectal cancer and esophageal adenocarcinoma. In certain subpopulations, insulin resistance, non-alcoholic liver disease, and neurodegenerative conditions have also been associated with *Hp*-I [10,11,12,13,14,15,16].

To date, *Hp* eradication treatment strategies have been revised in 2017 considering the increasing *Hp* antibiotic resistance. Quadruple regimens in high clarithromycin resistant regions in the absence of bismuth containing drugs were suggested [10]. Although clarithromycin resistance in Greece overwhelms the minimum acceptable value of 15% [10,17,18], many practitioners (especially non-gastroenterologists) prescribe outdated regimens such as a 10- or 14-day triple regimen. *Hp* eradication rates have been the most acceptable and reasonable way to compare regimens, although current financial circumstances require an additional economic approach to assist clinicians in the decision-making process. In this study, we aimed to estimate the direct costs of one attempt “outpatient” eradication treatment for all non-bismuth prescribed regimens in our country, considering the prices of both prototypes and generics.

## 2. Materials and Methods

**Figure 1:**
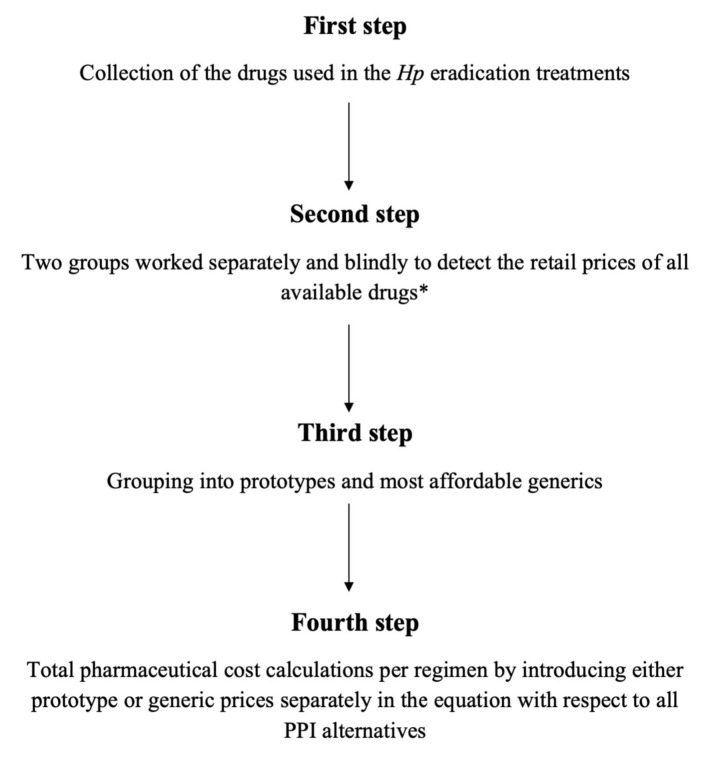
The four step working pathway to calculate the direct aggregated pharmaceutical costs for one attempt “outpatient” non-bismuth eradication therapies of *Helicobacter pylori* (*Hp*). All prototypes and generics drugs included in both first- and second-line treatment regimens were collected to calculate the overall cost per therapeutic choice. * Data regarding drug prices were collected and double-checked using two databases; a governmental website of the Greek National Organization for Medicines (https://www.eof.gr/web/guest/home) and a commercial, routinely used medical platform (https://www.galinos.gr/). PPI: proton pump inhibitor.

**Figure 2:**
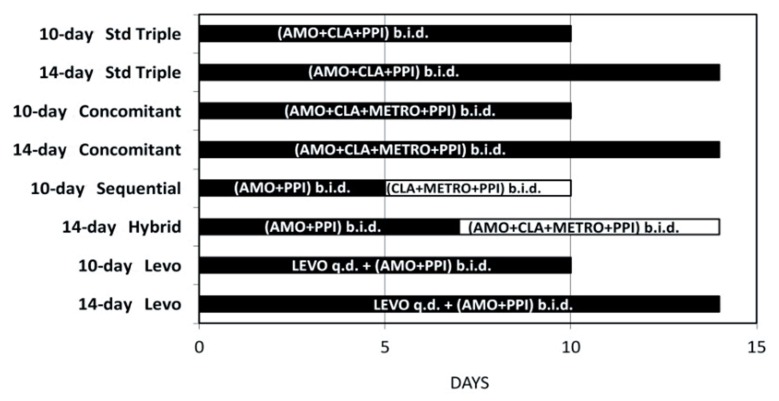
Detailed outline of first- and second-line *Hp-*I eradication regimens. Ten- and 14-day standard triple treatment includes PPI twice a day (b.i.d.), amoxicillin 1 g b.i.d., and clarithromycin 500 mg b.i.d. for ten and fourteen days, respectively. The concomitant regimen includes a continuous intake of PPI b.i.d., amoxicillin 1 g b.i.d., clarithromycin 500 mg b.i.d. and metronidazole 500 mg b.i.d. for either ten or fourteen days. Patients treated with the 10-day sequential regimen received amoxicillin 1 g b.i.d. and PPI b.i.d. for five days, followed by clarithromycin 500 mg b.i.d., metronidazole 500 mg b.i.d. and PPI b.i.d. for an additional five days. Regarding the 14-day hybrid regimen, the patients received PPI and amoxicillin 1 g for 14 days, with the addition of 500 mg clarithromycin and 500 mg metronidazole for the final seven days, all b.i.d.. In case of failure, second line rescue treatment involves the concomitant administration of levofloxacin 500 mg once a day (q.d.) plus amoxicillin 1 g b.i.d. and PPI b.i.d. for 10 or 14 days. AMO; amoxicillin 1 g, CLA; clarithromycin 500 mg, METRO; metronidazole 500 mg, PPI; proton-pump inhibitor, LEVO; levofloxacin 500 mg.

In order to calculate the direct aggregated pharmaceutical costs for one attempt “out-patient” non-bismuth eradication therapies for *Hp* we worked in four steps, as presented in Figure 1. A wide spectrum of potential *Hp* eradication treatment regimens were included in our survey (Figure 2):

- First-line regimens:
  i. 10-day standard triple
  ii. 14-day standard triple,
  iii. 10-day sequential,
  iv. 14-day sequential,
  v. 10-day concomitant non-bismuth quadruple
  vi. 14-day concomitant non-bismuth quadruple,
  vii. 14-day hybrid,
- Second-line salvage regimens:
  i. 10-day levofloxacin-containing triple regimen,
  ii. 14-day levofloxacin-containing triple regimen.

Initially, the active substances in the regimens were collected, including the choices of PPIs. Amoxicillin 1 g, clarithromycin 500 mg, metronidazole 500 mg and levofloxacin 500 mg constitute the mainstay antibiotics composing the various eradication regimens; levofloxacin has been considered as a component of the second-line salvage eradication regimen. The second pillar of *Hp-*I eradication treatment, complementary to antibiotics, is based on the anti-secretory and anti-inflammatory action of PPIs such as omeprazole 20 mg, esomeprazole 20 mg and 40 mg, rabeprazole 30 mg, pantoprazole 40 mg and lansoprazole 30 mg, which were included in our cost-calculating combinations. In our country, a chewable form of lansoprazole 30 mg is available and was also included.

In the next two steps, two separate study subgroups worked simultaneously but independently to detect the market prices of the aforementioned drug packages (“boite”) both for prototypes and the most affordable generics by searching in two different sources. Two official online medical databases, including all medicines approved by the Greek National Organization for Medicines, were conscripted and the results were compared and verified. The first database was the official government website of the Greek National Organization for Medicines and the second was a commercial medical platform routinely used by healthcare professionals in Greece [19,20]. All performed calculations referred to the current drug retail price configuration.

Finally, each drug’s cost was introduced in a summary equation per eradication treatment regimen including all used medications: Regimen’s cost *= aDrug*_1_ *+ bDrug*_2_ *+ … + zDrug_ν_;* with *Drug*_1_*, …, Drug_ν_*; the price of one “boite” of every used drug and *a,b, …, z*; the quantity of the medication’s packages.

Every regimen’s cost was calculated by introducing prototype or generic prices in the equation separately. All PPI alternatives per regimen were estimated to evaluate immiscible costs, comparing for both prototypes and generics.

Finally, additional costs of esophagoduodenoscopy, including biopsies, C^13^ Urea Breath Test (Helicobacter INFAI^®^Test) and serum anti-Hp antibody detection were calculated separately, as they were constant regardless of the treatment choice.

The study protocol was not sent for approval by the ethics committees of the involved hospitals as no patients were involved.

## 3. Results

One hundred and seventeen different values regarding *Hp* eradication treatment costs ensued and Table 1 summarizes these results. As expected, the market price of PPIs constituted the unique independent variable forming the total regimen cost in every therapeutic strategy; eradication treatment regimens including pantoprazole 40 mg appeared to have the lowest prices both in the prototype and generic groups. Moreover, regimens based on generics were more affordable than the respective prototypes.

Second-generation PPI use (esomeprazole, rabeprazole) burdened the regimens’ costs when compared to pantoprazole prescription. More specifically, an additional cost of 4.65 €, 5.79 € or 6.47 € per patient was added to the prototype eradication regimen using esomeprazole 20 mg, esomeprazole 40 mg or rabeprazole 30 mg, respectively, instead of pantoprazole 40 mg, whereas the analogous differences when using generics were 1.94 €, 2.79 € and 3.81 €, respectively. 

Furthermore, our analysis showed that omeprazole 20 mg generics were the most expensive among all PPI generics.

Additionally, the prescription of prototype regimens containing chewable lansoprazole 30 mg proved to be the most expensive pharmaceutical choice, adding a cost of 15.53 € to 23.38 € when compared to the most affordable alternative. Differences between the lowest and the highest cost per regimen are illustrated in Table 2.

Paradoxically, the 10-day concomitant as well as the 14-day hybrid regimens, although differing in duration and medication doses, costed equally. Finally, the 14-day triple regimen was more expensive than the aforementioned regimens, which are suggested as the most appropriate in our region.

Table 3 describes the non-pharmaceutical costs of *Hp*-I management.

## 4. Discussion

To our knowledge, this is the first study that has summarized and compared the total pharmaceutical costs of approved and routinely prescribed *Hp* eradication regimens and PPI variants based on the current market retail prices. Recently, De Francesco et al. [21] presented a relative comparison between regimens used in Italy but did not estimate the different costs between prototypes and generics or PPI alternatives. In addition, in our study, a direct comparison referring to medication prices was performed between prototype- and generic-based eradication regimens. As expected, the generic-based alternatives were more affordable than prototypes per regimen, and those using pantoprazole 40 mg as a PPI had the lowest price. Additionally, 10-day concomitant and 14-day hybrid eradication regimens, both with acceptable eradication rates in Greece, necessitate equal expenditures [17,22,23].

High antibiotic resistance has provoked a gradual escalation of eradication treatment regimens, following an “add-on” strategy, especially in regions such as in Greece where bismuth containing medications are not commercially available [18,24,25]. *Hp* resistance to clarithromycin (>20%) has been recognized as the most determinant hindrance of eradication treatment success [26] and in that setting, quadruple regimens are indicated [10,27]. However, in Greece, despite clear statements in current guidelines, some practitioners (especially non-gastroenterologists) insist on prescribing triple regimens. The 10-day triple regimen, despite being more affordable, yields unacceptable eradication rates in Greece [28,29] and the 14-day triple regimen, which only slightly increases effectiveness, consumes more funds compared to the indicated concomitant and hybrid schemes. Recent studies suggest that the 10-day concomitant and the 14-day hybrid are the most appropriate and effective regimens against *Hp-*I in the Greek environment when compared with the 10-day sequential treatment [17,22,23]. More specifically, eradication rates of 90.6–93.4% and 90.2% were yielded using the 10-day concomitant and the 14-day hybrid regimens, respectively [17,22,23]. Both schemes were found economically equal in our study, thus providing clinicians the flexibility to choose the most suitable for each patient, targeting best compliance. This paradox can be explained by considering the national policy on drug disposal. Pharmacies provide drugs in packages instead of the precise necessary quantity. Regarding the 10-day concomitant regimen, 20 tablets of amoxicillin 1 g are needed, whereas for the 14-day sequential, 28 are needed. Given that one package of amoxicillin 1 g contains 18 tablets, patients receive two packages (36 tablets) irrespective of the prescribed regimen. It should be noted that the 10-day concomitant regimen seems to achieve slightly higher eradication rates than the hybrid one and therefore further evaluation and cost–benefit analyses are necessary, including multiple variables, such as adverse effect expenditures, hospitalizations and *Hp-*I complication costs to draw conclusions regarding the most appropriate scheme.

A financial comparison between prototype- and generic-based regimens has been conducted in our study, although the results were what we expected. Despite the fact that generic drugs are generally more affordable, since recently, medical practitioners in Greece had been routinely prescribing prototypes [4]. An absence of clinical studies surveying the theoretical equality between prototypes and generics resulted in a subconscious doubt regarding effectiveness and/or safety of generics not only among doctors, but also among patients (at least the most *“*suspicious*”* of them) [5]. Furthermore, in Greece, there is lack of a disincentive for prescribing prototypes instead of generics, as the extra cost for the patient is actually negligible. The economic difference between the most affordable prototype-based 10-day concomitant eradication regimen and the respective generic one was 6.94 €. In concordance with government policy, patients are charged 25% of the prescription value, i.e., an extra cost of only 1.74 €. It is obvious that actions are needed, both in the form of disincentives for prescribing prototypes as well as studies on the efficacy and safety of generics.

A further novelty of our study was the direct economic comparison between PPI alternatives, although controversial data are reported regarding the most appropriate PPI component of *Hp* eradication schemes [29,30]. New generation PPIs, especially esomeprazole, have been proposed to be more effective than first generation options, although distinct pathophysiological mechanisms (such as CYP2C19 polymorphisms) are not yet clear [29,30]. Georgopoulos et al. and Apostolopoulos et al., although not performing a head-to-head comparison between different PPIs, presented slightly different eradication rates between esomeprazole 40 mg and pantoprazole 40 mg based schemes in the same population, implying esomeprazole superiority [17,22]. More specifically, the per protocol eradication rates of esomeprazole 40 mg based 10-day concomitant and 10-day sequential regimens were 93.4% and 82.8%, whereas the corresponding percentages using pantoprazole 40 mg were 90.6% and 78.1% respectively [17,22]. With regard to pharmaceutical costs, our study focused on the comparison between eradication regimens differing in PPIs, with pantoprazole 40 mg based regimens being the most affordable, combined with either prototypes or generics. Consequently, PPIs determined the overall costs of eradication regimens both in prototype and generic subgroups. When applying a classification from the lowest to the highest total price for any regimen, generic-based regimens using pantoprazole 40 mg were the most affordable, followed by esomeprazole 20 mg, esomeprazole 40 mg, rabeprazole 30 mg, lansoprazole 30 mg and omeprazole 20 mg generic regimens. Regarding prototypes regimens, pantoprazole 40 mg, esomeprazole 20 mg, esomeprazole 40 mg, rabeprazole 30 mg, omeprazole 20 mg, lansoprazole 30 mg and finally chewable lansoprazole 30 mg were the most expensive alternatives. The plethora of PPI choices requires clarification of their individual role in *Hp* eradication concerning and comparing not only their clinical effectiveness, but also their cost effectiveness.

The main limitation of our study was the isolated economical evaluation of *Hp*-I eradication treatment without performing multivariate cost–benefit or cost-utility analyses to compare the available eradication regimens. Nevertheless, the current study provided the initial stimulus suggesting further prospective national studies to provide a national consensus based on eradication rates, antibiotic resistance and cost effectiveness, especially after the dynamic alterations of the Greek population during the recent migratory flows [31,32,33,34]. As bismuth-based regimens are not commercially available in our country, we could not collect financial data and include them in our study. Additionally, a wide spectrum of *Hp*-I management costs needs to be considered in further studies, including diagnostic tests, potential eradication failures and second line treatment costs, hospitalizations, complications and any expenditure resulting from disorders related to *Hp*-I and its eradication treatment. Finally, a snapshot of drug retail prices has been used and fluctuations could not be predicted in this study. Therefore, a constant evaluation of treatment costs is necessary to secure a national management of healthcare funds.

## 5. Conclusions

In conclusion, this is the first study, at least in our country, evaluating and comparing *Hp* eradication treatment regimens from an economical aspect. Generic-based regimens (and more specifically those using pantoprazole as a PPI) achieve the lowest prices and national studies comparing the efficacy of generics and prototypes seem to be necessary. Our data should be taken into account by public health policymakers and physicians both in Greece and the European Union, aiming for a better and less expensive therapeutic approach. In Greece, during the financial crisis, healthcare policymakers have applied a restriction policy of healthcare expenditures to the detriment of long-term public health. Nowadays, cost–utility analyses appear more than necessary to promote a combined patient-centered and cost-restricting healthcare policy not only for *Hp-*I eradication, but also for every disease.

## Figures and Tables

**Table 1:** Total pharmaceutical costs per *Hp* non-bismuth containing eradication regimen using prototype or generic drug choices and different PPIs.

Eradication Treatment Regimens	Pharmaceutical Cost per Regimen in Euros (€)													
	Prototype-Based Regimens *							Generic-Based Regimens *						
	O	E (20 mg)	E (40 mg)	P	R	L	CL	O	E (20 mg)	E (40 mg)	P **	R	L	CL
**First Line Regimens**														
10-day standard triple	37.77	32.74	33.86	28.09	34.56	38.28	38.59	26.35	23.09	23.94	21.15	24.98	26.30	-
14-day standard triple	54.77	49.74	50.86	45.09	51.56	55.28	55.59	37.41	34.15	35.00	32.21	36.04	37.36	-
10-day sequential	38.57	33.54	34.66	28.89	35.36	39.08	39.39	27.15	23.89	24.74	21.95	25.78	27.10	-
14-day sequential	54.00	46.96	48.52	40.45	49.50	54.71	55.15	38.01	33.45	34.64	30.73	36.09	37.94	
10-day concomitant non-bismuth quadruple	42.55	37.52	38.64	32.87	39.34	43.06	43.37	31.13	27.87	28.72	25.93	29.76	31.08	-
14-day concomitant non-bismuth quadruple	59.55	54.52	55.64	49.87	56.34	60.06	60.37	42.19	38.93	39.78	36.99	40.82	42.14	-
14-day hybrid	42.55	37.52	38.64	32.87	39.34	43.06	43.37	31.13	27.87	28.72	25.93	29.76	31.08	-
**Second Line Salvage Regimens**														
10-day levofloxacin-containing triple regimen	31.03	26.00	27.12	21.35	27.82	31.54	31.85	21.16	17.90	18.75	15.96	19.79	21.11	-
14-day levofloxacin-containing triple regimen	36.16	31.13	32.15	26.48	32.95	36.67	36.98	25.21	21.95	22.80	20.01	23.84	25.16	-

* Prototype and generic drug subgroups include prototype and generic antibiotics respectively; ** Regimens with generics and pantoprazole are the most affordable treatment choice for *Hp* eradication. O: Omeprazole, E: Esomeprazole, P: Pantoprazole, R: Rabeprazole, L: Lansoprazole, CL: chewable Lansoprazole.

**Table 2:** Mean cost per eradication treatment regimen and price difference between the most expensive choice * and the most affordable one **.

Eradication Treatment Regimens	Mean Cost in Euros (€)	Price Difference in Euros (€)
**First Line Regimens**		
10-day standard triple	29.98	17.44
14-day standard triple	44.24	23.38
10-day sequential	30.78	17.44
14-day sequential	43.09	24.42
10-day concomitant non-bismuth quadruple	34.76	17.44
14-day concomitant non-bismuth quadruple	49.02	23.38
14-day hybrid	34.76	17.44
**Second Line Salvage Regimens**		
10-day levofloxacin-containing triple regimen	23.72	15.89
14-day levofloxacin-containing triple regimen	28.58	16.96

* The most expensive choice includes prototypes containing chewable lansoprazole; ** the most affordable choice includes generics containing pantoprazole.

**Table 3:** Non-pharmacological costs of *Hp*-I diagnostic evaluations *.

Intervention	Cost in Euros (€)
Esophagogastroduodenoscopy	77
Serum anti-*Hp* antibodies	13.60
C^13^ Urea Breath Test (Helicobacter Test INFAI^®^)	30.36
Gastric histology using Giemsa stain	27.76

* Based on the resource costs supplied by the Greek National Organization for Health Care Benefits Provision, *Hp*-I; Helicobacter pylori infection.
